# Health-related quality of life in glioma patients in China

**DOI:** 10.1186/1471-2407-10-305

**Published:** 2010-06-18

**Authors:** Jin-xiang Cheng, Bo-lin Liu, Xiang Zhang, Wei Lin, Yong-qiang Zhang, Wei-ping Liu, Jian-ning Zhang, Hong Lin, Rui Wang, Hong Yin

**Affiliations:** 1 Department of Neurosurgery, Xijing Institute of Clinical Neuroscience, Xijing Hospital, Fourth Military Medical University, Xi'an, Shaanxi Province, China; 2 Department of Health Statistics, Fourth Military Medical University, Xi'an, Shaanxi Province, China; 3 Department of Radiology, Xijing Hospital, Fourth Military Medical University, Xi'an, Shaanxi Province, China

## Abstract

**Background:**

Health-related quality of life (HRQOL) has been increasingly emphasized in cancer patients. There are no reports comparing baseline HRQOL of different subgroups of glioma patients prior to surgery.

**Methods:**

HRQOL assessments by the standard Chinese version of the European Organization for Research and Treatment of Cancer Quality of Life Core Questionnaire 30 (EORTC QLQ-C30, version 3.0), the Mini-Mental State Examination and Karnofsky Performance Status were obtained from glioma patients prior to surgery.

**Results:**

Ninety-two pathologically confirmed glioma patients were recruited. There were 84.8% patients with emotional impairment, 75% with social and cognitive impairment, 70.7% with physical impairment, and 50% with role impairment. Eighty-two percent of patients reported fatigue symptoms, 72.8% reported pain, 50% reported appetite loss, 39.1% reported insomnia, and 36.9% reported nausea/vomiting, whereas other symptoms (dyspnea, diarrhea, constipation) in the QLQ-C30 were reported by fewer than 30% of patients. Fatigue and pain symptoms and all "functioning" scales were strongly correlated with global health status/quality of life (QoL). Fatigue was strongly related to all functioning scales, pain, appetite loss, and global health status/QoL. No difference in baseline HRQOL prior to surgery was reported between females and males, among different lesion locations, or between normal- and abnormal-cognition subgroups of glioma patients. Age, KPS, WHO grade, and tumor recurrence significantly affected HRQOL in glioma patients.

**Conclusions:**

These data provided the baseline HRQOL in glioma patients prior to surgery in China. Most pre-surgery glioma patients indicated emotional, social, cognitive, physical, and role impairment. Fatigue, pain, appetite loss, insomnia, and nausea/vomiting were common in these patients. The fatigue and pain symptoms and all types of functioning strongly affected global health status/QoL. Old age, worse performance status, WHO grade IV and tumor recurrence had deleterious effects on HRQOL.

## Background

Cancers of the brain and nervous system account for 189,000 new cases and 142,000 deaths annually (1.7% of new cancers and 2.1% of cancer deaths), although such incidences are probably considerably underestimated because of the lack of sophisticated diagnostic technology [1]. Gliomas are the most common primary central nervous system (CNS) tumors; they account for over 40% of CNS tumors and 78% of CNS malignancies in adults [2]. Gliomas comprise a variety of histopathologic subtypes arising from the glial matter surrounding neurons in the brain, with different prognoses. The median survival is only 12-15 months for patients with glioblastomas (GBMs) (WHO grade IV), 2-5 years for patients with anaplastic gliomas (WHO grade III) [3] and 4-10 years for patients with low-grade gliomas (LGG, including WHO grades I and II) [4-6]. Survival is often limited by tumor recurrence and progression of LGG to high-grade gliomas (HGG, including WHO grades III and IV). Factors influencing survival in these patients include histologic subtype, lesion location, age, KPS and cognitive function [7,8]. Maximal resection [3,9,10], radiotherapy [11-13], and chemotherapy [12,13] are reported to be associated with longer overall survival. There is no consensus on the optimal management of patients with residual LGG and HGG following surgical resection, and even whether (and to what extent) surgical resection leads to improvements in patient outcome and survival has been questioned by some neurologists [14,15]. During recent decades, many studies evaluating new therapeutic protocols for cancer patients mainly considered overall survival and progression-free survival as primary response measures. It is increasingly recognized that the choice of therapeutic strategy also should entail careful consideration of its effects on the quality of life (QoL) during the remaining survival time. Recent research on health-related quality of life (HRQOL) of patients with gliomas has been conducted to evaluate the effects of new treatments in English-speaking and western European countries. The European Organization for Research and Treatment of Cancer (EORTC) developed the Core Quality of Life Questionnaire (QLQ-C30) [16] for assessing the HRQOL in cancer patients and the Brain Cancer Module (BCM) [17] for brain tumor patients. The standard Chinese version of QLQ-C30 (version 3.0) is, overall, a valid instrument to assess HRQOL in Chinese breast, gynecological, and lung cancer patients [18], and its reliability and validity in brain tumor patients will be reported in another paper (unpublished data). To date, there is no Chinese version of BCM available. Osoba et al. reported the frequency of self-report symptoms and the effects of disease burden and neurological dysfunction on QoL in patients with HGG after surgery but prior to other adjuvant therapy [19,20]. Gustafsson et al. reported a cross-sectional study on QoL in LGG patients after various treatments using QLQ-C30 [6]. Findings from a meta-analysis of individual patient data from EORTC clinical trials have shown that baseline HRQOL parameters (physical functioning, appetite loss, and pain) post-surgery but before adjuvant treatment provide significant prognostic value in various cancer patients [21]. However, the baseline HRQOL in glioma patients prior to surgery is unknown, especially in China, where there have been no studies on HRQOL in glioma. The correlation between demographic and clinical variables and HRQOL pre-operation also remains elusive.

In this report, we presented baseline HRQOL measured by the Chinese version of QLQ-C30 (version 3.0) for glioma patients in China. We analyzed the relationship of demographic and clinical variables with HRQOL before surgery in glioma patients.

## Methods

### Subjects

This study was part of a preliminary study on HRQOL in brain tumor patients in China. The reliability and validity of the standard Chinese version of EORTC QLQ-C30 (version 3.0) and these patients' HRQOL at baseline (i.e., following diagnosis, prior to the start of surgery) will be reported in another article (unpublished data). In short, consecutive series of patients with either suspected brain tumor or diagnosed by CT or MRI were recruited from July 2008 to December 2008 in the Department of Neurosurgery, Xijing Institute of Clinical Neuroscience, Xijing Hospital, Fourth Military Medical University. The study was approved by the Institutional Review Board. Informed consent was obtained from all patients. No restrictions were placed on patient selection with regard to histologic type of brain tumors, age, education, cognitive function or performance status. The sample was restricted to patients who required operation. Post-operation patients with scheduled radiotherapy or chemotherapy were excluded. The standard Chinese version of QLQ-C30 (version 3.0) was administered following diagnosis but prior to operation or re-operation for all eligible patients. Sociodemographic and clinical data were recorded before treatment. Scoring of the Chinese version of the Mini-Mental State Examination (MMSE) [22] and Karnofsky Performance Status (KPS) [23] were performed by the doctors or nurses at the time of the first administration of the QLQ-C30.

### The Standard Chinese version of EORTC QLQ-C30 (version 3.0)

The EORTC QLQ-C30 (version 3.0) is a 30-item questionnaire composed of multi-item scales and single items that reflect the multidimensionality of the QoL construct. It combines five functional scales (physical, role, cognitive, emotional, and social), three symptom scales (fatigue, pain, and nausea/vomiting), a global health and QoL scale, six single items assessing additional symptoms commonly reported by cancer patients (dyspnea, appetite loss, sleep disturbance, constipation, and diarrhea), as well as the perceived financial impact of the disease and treatment [16,18].

### Analysis Plan

Only patients with pathologically confirmed glioma were included in the analysis. Based on MMSE scores, patients were divided into normal- and abnormal-cognition groups [22,24]. Briefly, MMSE scores less than 18 in illiterate patients, less than 21 in patients with elementary school education, and less than 25 in patients with more than high school education were defined as indicating abnormal cognition. Scoring of the responses to the QLQ-C30 (version 3.0) was carried out as previously described [25]. The raw scores for each domain and single item were transformed to give a value between 0-100. For the five functional scales and the global health status/QoL scale, item responses were recoded so that a higher score represented a better level of functioning. For the symptom-oriented scales and items, a higher score corresponded to a severe level of symptoms. Differences of at least 10 points (on a 0-100 scale) were classified as clinically meaningful changes in the mean value of a HRQOL parameter [26]. Descriptive analysis of HRQOL at the time of enrollment as measured by QLQ-C30 (version 3.0) was performed. Differences between or within subgroups at baseline with respect to each patient characteristic variable were assessed for all QoL subscales or items using the Mann Whitney U-test or Kruskal-Wallis test. Spearman's rank correlation was used to investigate relationships between the age, KPS, WHO grade and QLQ-C30 subscales and items. A chi-square test estimated the constituent ratio of MMSE scores between different age subgroups. A P value < .05 was considered statistically significant. Statistical analysis was performed using the SPSS software package, version 16.0 (SPSS Inc, Chicago, IL, USA).

## Results

### Patient Characteristics

In total, 308 brain tumor patients (excluding the pathologically confirmed non-cancer patients after surgery) were enrolled between July 2008 and December 2008, of whom 92 (29.87%) were pathologically confirmed glioma patients. Glioma patient characteristics at enrollment are shown in Table 1. Of the 92 patients evaluated, 57% were male, and the median age was 42 years (range, 4-76). The median KPS score was 90, and 21.74% of patients had abnormal cognition. There were 85.87% supratentorial tumors, of which 40.5% were located in the left hemisphere. There were 79 patients with newly diagnosed glioma and 13 patients with recurrent glioma. Table 2 shows the means, standard deviations, median and mode for the multi-item and single-item measures of the QLQ-C30. The proportions of all glioma patients with different scores at baseline prior to surgery in the functioning and symptom domains are shown in Additional file 1: Table S1. The full range of possible scores was observed for all measures except for emotional and cognitive functioning scales (range, 17-100). Score distributions were negatively skewed for all the functioning scales (i.e., more patients scored toward maximum functioning). The median values of social, emotional, cognitive, physical and role functioning were 66.7, 75.0, 83.3, 86.7 and 91.7, respectively. Of the patient, 84.8% reported emotional impairment, 75% reported social and cognitive impairment, 70.7% reported physical impairment, and 50% reported role impairment. Therefore, the patients reported more and severer difficulties in emotional and social domains than in cognitive, physical and role functioning. The median values were higher for symptoms of fatigue (33.3), pain (25) and appetite loss (16.7) than for others (0). Eighty-two percent of patients reported fatigue symptoms, 72.8% reported pain, 50% reported appetite loss, 39.1% reported insomnia, and 36.9% reported nausea/vomiting, whereas other symptoms (dyspnea, diarrhea, and constipation) in the QLQ-C30 were reported in less than 30% of all patients. For global health status/QoL, 47.8% patients reported scores of 50 or less. We further analyzed the relationships between the item-evaluating scales and single items in the QLQ-C30. The results revealed that there was a significant correlation between global health status/QoL and fatigue (r_s _= -.64, p < .001), physical functioning (r_s _= .62, p < .001), emotional functioning (r_s _= .57, p < .001), role functioning (r_s _= .57, p < .001), cognitive functioning (r_s _= .55, p < .001), pain (r_s _= -.56, p < .001), and social functioning (r_s _= .50, p < .001). In addition to global health status/QoL, fatigue was significantly correlated with physical functioning (r_s _= -.738, p < .001), cognitive functioning (r_s _= -.619, p < .001), role functioning (r_s _= -.631 p < .001), pain (r_s _= .59, p < .001), emotional functioning (r_s _= -.527, p < .001), appetite loss (r_s _= .50, p < .001) and social functioning (r_s _= -.457, p < .001).

**Table 1:** Patient characteristics at enrollment.

Characteristic	Number (%)
Gender	
Male	57(61.96%)
Female	35(38.04%)
Median age at diagnosis	42 (range, 3-76)
Tumor type	
A^	17(18.47)
OL^	7(7.61)
OA	9(9.78)
AA^	11(11.96)
AO^	2(2.17)
AOA	6(6.52)
GBM	34(36.96)
Other^§^	6(6.52)
WHO grade	
I	3(3.26)
II	35(38.04)
III	20(21.74)
IV	34(36.96)
Tumor location	
Sub	13(14.13)
Sup	79(85.87)
Frontal lobe	22(27.85)
Temporal lobe	21(26.58)
Parietal lobe	6(7.59)
Occipital lobe	1(1.27)
Multiple lobes	23(29.11)
Other	6(7.60)
Tumor hemisphere	
Left	32(40.50)
Right	44(55.70)
Both	3(3.80)
KPS	
100	4(4.3)
90	40(43.5)
80	20(21.7)
70	7(7.6)
60	5(5.4)
50	6(6.5)
<50	2(2.2)
Unknown	8(8.7)
Cognitive function	
Normal	71(77.17)
Abnormal	20(21.74)
Unknown	1(1.09)

^ 1 recurrent A, 1 recurrent AA, 1 recurrent AO, 1 recurrent OL.

* 6 recurrent GBM from de novo GBM, 2 secondary GBM.

^§^1 gangliocytoma WHO grade I, 1 choroid plexus papilloma WHO grade I, 1 anaplastic papillary glioneuronal tumor WHO grade III, 2 ependymoma WHO grade II, 1 recurrent pilocytic astrocytoma WHO grade I.

Abbreviations: A: astrocytoma; AA: anaplastic astrocytoma; AOA: anaplastic oligoastrocytoma; AO: anaplastic oligodendroglioma; GBM: glioblastoma; OA: oligoastrocytoma; OL: oligodendroglioma; Sub: subtentorial tumor; Sup: supratentorial tumor.

**Table 2:** Descriptive statistics of QLQ-C30 in glioma patients.

	Items*	Mean(SD)	Median	Mode
Functioning scales^				
PF	1-5	79.7(25.1)	86.7	100
RF	6, 7	75.9(30.2)	91.7	100
EF	21-24	75.8(18.6)	75.0	66.7
CF	20, 25	75.0(21.8)	83.3	83.3
SF	26, 27	69.2(26.7)	66.7	66.7
QL	29, 30	54.3(28.7)	58.3	83.3
Symptom scales and/or items§				
FA	10, 12, 18	34.1(25.1)	33.3	33.3
NV	14, 15	14.1(24.9)	0.0	0
PA	9, 19	28.3(27.2)	25.0	33.3
DY	8	11.4(19.4)	0.0	0
SL	11	21.7(32.6)	0.0	0
AP	13	23.2(28.3)	16.7	0
CO	16	14.5(25.8)	0.0	0
DI	17	4.0(11.9)	0.0	0
FI	28	44.6(34.7)	33.3	33.3

* Numbers correspond to the item numbers in the questionnaire.

^ Scores range from 0 to 100, with a higher score representing a higher level of functioning.

§Scores range from 0 to 100, with a higher score representing a greater degree of symptoms.

Abbreviations: AP: appetite loss, CF: cognitive functioning; CO: constipation; DI: diarrhea; DY: dyspnea; EF: emotional functioning; FA: fatigue; FI: financial difficulties; L: left cerebral hemisphere; NV: nausea/vomiting; PA: pain; QL: global health status; SD: standard deviation; SF: social functioning; SL: insomnia

### HRQOL in different subgroup of glioma

There were no differences in baseline score in any QLQ-C30 subscale or item between female and male patients, between patients with subtentorial and supratentorial tumors, between patients with supratentorial tumors in the left and right hemispheres, among patients with tumors located in different lobes (frontal, temporal, parietal, multiple lobes), or between patients with normal and abnormal cognitive function (Additional file 2: Table S2). The frequencies of dyspnea, diarrhea and constipation are not shown in Additional file 2: Table S2 because of their low frequencies. The perceived financial impact of the disease and treatment is also not included in Additional file 2: Table S2.

Patients older than 50 reported worse physical (mean score 72.2 vs. 84.2, p = .014) and role functioning (66.7 vs. 81.6, p = .045), worse global health status/QoL (45.8 vs. 59.4, p = .035), and more insomnia (30.5 vs. 16.4, p = .047) and constipation (23.8 vs. 8.8, p = .002) symptoms than those younger than 50 (Additional file 2: Table S2). Though no differences were apparent on the cognitive functional scale, the proportion of patients diagnosed with abnormal cognition by MMSE was significantly larger in patients older than 50 than in patients younger than 50 (abnormal/abnormal cognition 12/22 vs. 8/49, p = .018). Patients with KPS less than 80 reported worse physical (51.3 vs. 89, p < .001), role (48.3 vs. 84.9, p < .001), emotional (65 vs. 80, p = .003), cognitive (55.8 vs. 81.3, p < .001), and social (55.8 vs. 73.7, p = .001) functioning, worse global health status/QoL (29.4 vs. 61.7, p < .001), and more symptoms of fatigue (60 vs. 25.7, p < .001), pain (45.8 vs. 22.9, p = .023), insomnia (38.3 vs. 15.6, p = .011) and appetite loss (41.7 vs. 17.7, p = .002) than those with KPS 80-100 (Additional file 2: Table S2). Abnormal cognition according to MMSE was associated with worse KPS compared with normal cognition (75 vs. 82, p = .043).

Patients with WHO grade I tumors were excluded from the analysis because of their low incidence. There was a trend that patients with grade IV tumors showed worse functioning, worse global health status/QoL, and more symptoms than patients with grade II and III tumors. Patients with grade IV tumors reported significantly worse physical (II, III, IV: 83.2, 90, 68.8, respectively; p = .005), role (80.5, 82.5, 66.2; p = .036), emotional (75.9, 85.8, 70.1; p = .007) and social (76.2, 77.5, 58.3; p = .004) functioning scores, worse global health status/QoL (58.1, 66.7, 40.6; p = .002,), and more fatigue (31.4, 20.6, 46.4; p < .001) and pain (28.1, 16.7, 36.8; p = .015) symptoms (Additional file 2: Table S2). Though no difference in the aforementioned scales was found between patients with grade II and III tumors, patients with grade III tumors reported better function and global health status/QoL and fewer symptoms than those with grade II tumors. Correlation analyses showed a significant relationship between KPS and physical functioning (r_s _= .61 p < .001), fatigue (r_s _= -0.53, p < .001), cognitive functioning (r_s _= .48 p < .001), role functioning (r_s _= .45 p < .001) and global health status/QoL (r_s _= .42 p < .001) in the QLQ-C30. Neither age nor WHO grade was related to any scale or item in the QLQ-C30.

Patients with recurrent glioma, most of which were recurrent from primary GBMs (6/13), showed worse physical and social function (71.3 vs. 81.1, p = 0.026 and 52.6 vs. 71.9, p = 0.019, respectively) and worse global health status/QoL (39.7 vs. 56.7, p = .047) than newly diagnosed glioma patients (Additional file 2: Table S2).

## Discussion

Despite efforts made by neuro-oncologists over the past several decades, the management of CNS tumors remains a highly challenging task, with few improvements in long-term survival, especially in WHO grade III and IV malignancies. Under these circumstances, the HRQOL is a very important consideration in the overall management of these patients. Very few data are available about the HRQOL of patients with glioma prior to surgery, while more data are available regarding HRQOL in glioma patients after surgery but before adjuvant therapy, mainly due to analyses of a large database of patients preparing to undergo chemo- or radio-therapy [19,20,27-29]. Osoba et al. [19] analyzed the differences in patients' HRQOL at recurrence compared to newly diagnosed disease, the frequency and severity of symptoms (symptom burden), and the effects of tumor histology and baseline HRQOL of patients with HGG compared to patients with other types of cancer. In summary, six symptoms (fatigue, uncertainty about the future, motor difficulties, drowsiness, communication difficulties, and headache) were reported with a frequency >50% by both GBM and anaplasia astrocytoma (AA) patients; visual problems and pain symptoms were also reported with frequencies of >50% by patients with recurrent GBM; problems with motor functioning, vision, leg strength, and pain were reported more frequently by patients with recurrent GBM than by those with recurrent AA; scores on HRQOL functioning scales were similar in GBM and AA groups; the QLQ-C30 scores for HGG patients were similar to those with metastatic cancers and worse than those with localized cancers [19]. A cross-sectional study on HRQOL in LGG patients after various treatments, conducted by Gustafsson et al.[6], found that nearly all patients were capable of self-care, but less than half were able to carry out normal activities without restriction. The most frequent symptoms were fatigue, sleep disturbances and pain, while the most difficult types of functioning were role, cognitive and emotional functioning. Of the patients, 45% had scores indicating low overall QoL, and mental problems had a stronger impact on QoL than physical ones. A meta-analysis from EORTC confirmed that the baseline HRQOL post-surgery but before other adjuvant therapies was a prognostic factor for cancer survival [21].

In the current study, most glioma patients reported difficulties in emotional, social, cognitive, physical and role functioning. Glioma had more burden effects on patients' social and emotion functioning than other functioning. All of these functioning scales were correlated with global health status/QoL. Emotional dysfunction, such as depression, anxiety, nervousness and irritability, were common. Psychosocial intervention is needed for these patients. The most common symptoms in these patients were fatigue, pain, appetite loss, insomnia, and nausea/vomiting. Fatigue was strongly related to all functioning, pain, appetite loss and global health status/QoL. Therefore, it is important for clinicians to realize and manage fatigue. Compared with the study of Gustafsson et al. on long-term LGG patients [6], pre-surgery glioma patients in our study reported less cognitive impairment. Previous studies have found that radiotherapy, antiepileptic drugs and disease progression may cause cognitive impairment [30-33]. However, we need further studies to document the effects of therapies and diseases on HRQOL changes in newly diagnosed glioma patients before any treatment (including surgery), after treatment and during disease progression in a single cohort. Therefore, ways of avoiding cognitive impairment by the treatment should be taken into consideration for glioma patients. Compared with the reports from Osoba et al. [19,20] and Taphoorn et al. [28,29], patients in our study only completed the standard Chinese version of QLQ-C30 because the Chinese version of BCM20 was unavailable, which could have provided limited information on brain tumor-specific symptoms and functions. Patients in our study included those with newly diagnosed glioma prior to first surgery and recurrent glioma prior to re-operation. No differences in scales or items in QLQ-C30 were found between females and males or among patients with differently located lesions, which disagrees with some other reports [34,35]. It is difficult to compare these findings with our results, given the different measures that were used.

The MMSE is a well-validated and widely used screening test for dementia and cognitive impairment [9,13,30,36-38]. It was unexpected that no differences were found in any scale or item of QLQ-C30 (including cognitive functioning scale) between patients classified as having normal and abnormal cognition by MMSE. This result is discordant with the finding that in a cohort with various brain tumors, patients with normal cognition reporting better physical and cognitive functioning, better global health status/QoL, and worse fatigue and appetite loss symptom levels (unpublished data), and that deterioration in neurological function was accompanied by significant deterioration in several QoL domains and in global QoL [20]. We also found no difference in cognitive functioning between patients older versus younger than 50; however, there were more abnormally cognitive patients as tested by MMSE among patients older than 50. These discrepancies may be attributed to poor validation of the cognitive functioning scale itself in the standard Chinese version of QLQ-C30 [18,39] and to the possibility that more patients with cognitive dysfunction according to MMSE had their QLQ-C30 completed by their family members. Proxy-reported outcomes are often discrepant with patient-reported outcomes [40].

Patients with KPS 80-100 presented with better functioning and global health status/QoL and fewer fatigue, pain, insomnia and appetite loss symptoms than those with KPS less than 80. It is noteworthy that 69.2% of patients had KPS of 80-100, while no difference was shown in any scale or item of QLQ-C30 among these KPS subgroups (KPS 80, 90 and 100; data not shown). Correlational analysis showed that KPS was more strongly related to physical functioning but more weakly related to global health status/QoL. Additionally, most current research excluded patients with KPS less than 70 [11,19]. Therefore, these data may suggested that KPS was an inadequate surrogate for HRQOL. However, KPS may still be useful in studies of HRQOL among patients who are unable to provide reliable self-reported information.

There was a trend that patients with grade IV tumors showed worse functioning (except for cognitive functioning), worse global health status/QoL, and more fatigue and pain symptoms than patients with grade II and III tumors. However, no statistically significant differences existed between patients with grade II and III tumors. On the other hand, Gustafsson et al. compared the HRQOL outcomes in LGG (WHO grades I and II) patients with those of HGG (WHO grades III and IV) patients reported by Osoba et al. and found that the LGG patients reported better physical, role and social functioning and less fatigue and nausea, but they had more pain, with similar cognitive and emotional functioning, global health status/QoL and other symptoms [6]. The causes of this disparity are unknown. It might be postulated that WHO grade II patients showed more dysfunction and symptoms prior to surgery, which led them to early treatment and thus a better prognosis and HRQOL after surgery than WHO grade III patients. Another explanation could be that patients had their poor prognoses of WHO grade III or their better prognoses of WHO grade II confirmed after surgery, and this knowledge may psychologically affect their assessments of QoL. Patients with recurrent glioma showed worse physical and social functioning and global health status/QoL, as cancer recurrence may contribute to HRQOL deterioration.

Our study has several limitations. First, we did not use the BCM, as there was no available Chinese version. Using the BCM would have allowed us to evaluate many brain tumor-specific symptoms. Future studies should include brain tumor-specific instruments. Second, larger samples are required to validate our results. Third, the study was cross-sectional and had a descriptive and correlative design. Further research should follow these patients and record their HRQOL changes during the whole disease course.

## Conclusions

In summary, this report provided the baseline HRQOL of 92 glioma patients prior to surgery in China. Most glioma patients prior to surgery presented with emotional, social, cognitive, physical and role impairments. Fatigue, pain, appetite loss, insomnia, and nausea/vomiting were common in these patients. The fatigue and pain symptoms and all scales of functioning were strongly correlated with global health status/QoL. Fatigue was strongly related to all functioning scales, pain, appetite loss, and global health status/QoL. Clinicians should help patients deal with these symptoms and improve functioning. Old age, worse performance status, WHO grade IV and tumor recurrence had deleterious effects on HRQOL.

## Abbreviations

A: astrocytoma; AA: anaplastic astrocytoma; Ab: abnormal cognition; AO: anaplastic oligodendroglioma; AOA: anaplastic oligoastrocytoma; AP: appetite loss; BCM: Brain Cancer Module; CF: cognitive functioning; CO: constipation; DI: diarrhea; DY: dyspnea; EF: emotional functioning; EORTC: European Organisation for Research and Treatment of Cancer; F: female; FA: fatigue; FI: financial difficulties; GBM: glioblastoma; HGG: high-grade gliomas; HRQOL: health-related quality of life; KPS: Karnofsky Performance Status; L: left cerebral hemisphere; LGG: low-grade gliomas; M: male; MMSE: Mini-Mental State Examination; MR: mean rank; N: normal cognition; NV: nausea/vomiting; OA: oligoastrocytoma; OL: oligodendroglioma; PA: pain; QL: global health status; QLQ-C30: Quality of Life Core Questionnaire 30; R: right cerebral hemisphere; SF: social functioning; SL: insomnia; Sub: subtentorial tumor; Sup: supratentorial tumor.

## Competing interests

The authors declare that they have no competing interests.

## Authors' contributions

JXC and BLL participated in the design of the study, administered the questionnaire, maintained case records, and drafted the manuscript. XZ and WL participated in the design of the study, administered the questionnaire, and revised the manuscript. YQZ and RW participated in the design of the study and performed the statistical analysis. WPL and JNZ conceived of the study, and participated in its design and coordination. HL and HY participated in the radiologic diagnosis and coordination. All authors read and approved the final manuscript.

## Pre-publication history

The pre-publication history for this paper can be accessed here:

http://www.biomedcentral.com/1471-2407/10/305/prepub

## Supplementary Material

Additional file 1 **Table S1**. Frequency of QLQ-C30 scores for all functioning and symptom scales and items in glioma patients. Click here for file

Additional file 2 **Table S2**. Comparisons of QLQ-C30 among different glioma subgroups. Click here for file
