# Supplementary material for: Health-related quality of life in glioma patients in China
Source: BMC Cancer. 2010 Jun 18;10:305. doi: 10.1186/1471-2407-10-305 (PMC2910687; doi:10.1186/1471-2407-10-305)
Supplement: Additional file 1 — Table S1. Frequency of QLQ-C30 scores for all functioning and symptom scales and items in glioma patients. [file 1471-2407-10-305-S1.DOC]

## Table S1. Frequency of QLQ-C30 scores for all functioning and symptom scales and items in glioma patients.

| PF | N | % |
| --- | --- | --- |
| 0.0 | 3 | 3.3 |
| 13.3 | 2 | 2.2 |
| 26.7 | 1 | 1.1 |
| 33.3 | 1 | 1.1 |
| 40.0 | 1 | 1.1 |
| 46.7 | 2 | 2.2 |
| 53.3 | 5 | 5.4 |
| 60.0 | 3 | 3.3 |
| 66.7 | 7 | 7.6 |
| 73.3 | 3 | 3.3 |
| 80.0 | 5 | 5.4 |
| 86.7 | 16 | 17.4 |
| 91.7 | 1 | 1.1 |
| 93.3 | 15 | 16.3 |
| 100.0 | 27 | 29.3 |
| Total | 92 | 100 |
|  |  |  |
| RF | N | % |
| 0.0 | 5 | 5.4 |
| 16.7 | 3 | 3.3 |
| 33.3 | 7 | 7.6 |
| 50.0 | 3 | 3.3 |
| 66.7 | 23 | 25 |
| 83.3 | 5 | 5.4 |
| 100.0 | 46 | 50.0 |
| Total | 92 | 100 |
|  |  |  |
| EF | N | % |
| 16.7 | 1 | 1.1 |
| 33.3 | 3 | 3.3 |
| 41.7 | 3 | 3.3 |
| 50.0 | 4 | 4.3 |
| 55.6 | 1 | 1.1 |
| 58.3 | 7 | 7.6 |
| 66.7 | 15 | 16.3 |
| 75.0 | 15 | 16.3 |
| 83.3 | 14 | 15.2 |
| 91.7 | 15 | 16.3 |
| 100.0 | 14 | 15.2 |
| Total | 92 | 100 |
|  |  |  |
| CF | N | % |
| 16.7 | 3 | 3.3 |
| 33.3 | 6 | 6.5 |
| 50.0 | 7 | 7.6 |
| 66.7 | 25 | 27.2 |
| 83.3 | 28 | 30.4 |
| 100.0 | 23 | 25.0 |
| Total | 92 | 100 |
|  |  |  |
| SF | N | % |
| 0.0 | 4 | 4.3 |
| 16.7 | 1 | 1.1 |
| 33.3 | 11 | 12.0 |
| 50.0 | 9 | 9.8 |
| 66.7 | 26 | 28.3 |
| 83.3 | 18 | 19.6 |
| 100.0 | 23 | 25.0 |
| Total | 92 | 100 |
|  |  |  |
| QL | N | % |
| 0.0 | 5 | 5.6 |
| 8.3 | 4 | 4.4 |
| 16.7 | 8 | 8.9 |
| 25.0 | 1 | 1.1 |
| 33.3 | 7 | 7.8 |
| 41.7 | 11 | 12.2 |
| 50.0 | 7 | 7.8 |
| 58.3 | 4 | 4.4 |
| 66.7 | 14 | 15.6 |
| 75.0 | 6 | 6.7 |
| 83.3 | 16 | 17.8 |
| 100.0 | 7 | 7.8 |
| Total | 90 | 100 |
|  |  |  |
| FA | N | % |
| 0.0 | 11 | 12.0 |
| 11.1 | 16 | 17.4 |
| 22.2 | 11 | 12.0 |
| 33.3 | 21 | 22.8 |
| 44.4 | 13 | 14.1 |
| 55.6 | 8 | 8.7 |
| 66.7 | 2 | 2.2 |
| 77.8 | 5 | 5.4 |
| 88.9 | 3 | 3.3 |
| 100.0 | 2 | 2.2 |
| Total | 92 | 100 |
|  |  |  |
| NV | N | % |
| 0.0 | 59 | 64.1 |
| 16.7 | 12 | 13.0 |
| 33.3 | 12 | 13.0 |
| 50.0 | 2 | 2.2 |
| 66.7 | 3 | 3.3 |
| 100.0 | 4 | 4.3 |
| Total | 92 | 100 |
|  |  |  |
| PA | N | % |
| 0.0 | 25 | 27.2 |
| 16.7 | 21 | 22.8 |
| 33.3 | 29 | 31.5 |
| 50.0 | 3 | 3.3 |
| 66.7 | 7 | 7.6 |
| 83.3 | 2 | 2.2 |
| 100.0 | 5 | 5.4 |
| Total | 92 | 100 |
|  |  |  |
| DY | N | % |
| 0.0 | 64 | 70.3 |
| 33.3 | 24 | 26.4 |
| 66.7 | 2 | 2.2 |
| 100.0 | 1 | 1.1 |
| Total | 91 | 100 |
|  |  |  |
| SL | N | % |
| 0.0 | 56 | 60.9 |
| 33.3 | 21 | 22.8 |
| 66.7 | 6 | 6.5 |
| 100.0 | 9 | 9.8 |
| Total | 92 | 100 |
|  |  |  |
| AP | N | % |
| 0.0 | 46 | 50.0 |
| 33.3 | 33 | 35.9 |
| 66.7 | 8 | 8.7 |
| 100.0 | 5 | 5.4 |
| Total | 92 | 100 |
|  |  |  |
| CO | N | % |
| 0.0 | 65 | 70.7 |
| 33.3 | 17 | 18.5 |
| 66.7 | 7 | 7.6 |
| 100.0 | 3 | 3.3 |
| Total | 92 | 100 |
|  |  |  |
| DI | N | % |
| 0.0 | 82 | 89.1 |
| 33.3 | 9 | 9.8 |
| 66.7 | 1 | 1.1 |
| Total | 92 | 100 |
|  |  |  |
| FI | N | % |
| 0.0 | 22 | 23.9 |
| 33.3 | 34 | 37.0 |
| 66.7 | 19 | 20.7 |
| 100.0 | 17 | 18.5 |
| Total | 92 | 100 |

Abbreviations: AP: appetite loss, CF: cognitive functioning; CO: constipation; DI: diarrhea; DY: dyspnea; EF: emotional functioning; FA: fatigue; FI: financial difficulties; L: left cerebral hemisphere; NV: nausea/vomiting; PA: pain; QL: global health status; SF: social functioning; SL: insomnia
