# Supplementary material for: Health-related quality of life in glioma patients in China
Source: BMC Cancer. 2010 Jun 18;10:305. doi: 10.1186/1471-2407-10-305 (PMC2910687; doi:10.1186/1471-2407-10-305)
Supplement: Additional file 2 — Table S2. Comparisons of QLQ-C30 among different glioma subgroups. [file 1471-2407-10-305-S2.DOC]

## Table S2. Comparisons of QLQ-C30 among different glioma subgroups.

* p < .05; ** p< .01

Abbreviations: Ab: abnormal cognition; AP: appetite loss, CF: cognitive functioning; CO: constipation; DI: diarrhea; DY: dyspnea; EF: emotional functioning; F: female; FA: fatigue; FI: financial difficulties; L: left cerebral hemisphere; M: male; MR: mean rank; N: normal cognition; NV: nausea/vomiting; New: new diagnosed glioma; PA: pain; QL: global health status; R: right cerebral hemisphere; Re: recurrent glioma; SF: social functioning; SL: insomnia; Sub: subtentorial tumor; Sup: supratentorial tumor.

|  |  | PF | EF | CF |
| --- | --- | --- | --- | --- |
| Age  (<50y/≥50y) | Mean | 84.3/72.2 | 77.9/72.3 | 76.3/72.9 |
|  | MR | 51.8/37.9^*^ | 49.6/41.5 | 48.4/43.4 |
|  | p | .014 | .155 | .360 |
| Sex (F/M) | Mean | 80.1/79.4 | 74/76.9 | 72.9/76.3 |
|  | MR | 44.4/47.8 | 42.4/49 | 43.4/48.4 |
|  | p | .544 | .242 | .369 |
| Location (Sup/  Sub) | Mean | 79.6/80 | 76.3/72.4 | 74.3/79.5 |
|  | MR | 46.9/44.4 | 47.2/42.6 | 45.9/49.9 |
|  | p | .749 | .564 | .610 |
| Location (L/R) | Mean | 80/78.9 | 76.2/76 | 70.8/76.1 |
|  | MR | 40/37.4 | 39.5/37.8 | 37/39.6 |
|  | p | .607 | .734 | .602 |
| Cognition (N/Ab) | Mean | 81/78.3 | 75.9/75.3 | 76.8/69.2 |
|  | MR | 47.2/41.7 | 46.1/45.5 | 48.1/38.5 |
|  | p | .398 | .923 | .135 |
| KPS  (<80/≥80) | Mean | 51.3/89 | 65/80 | 55.8/81.3 |
|  | MR | 15.1/51.1^**^ | 28.3/47^**^ | 21.8/49^**^ |
|  | p | .000 | .003 | .000 |
| WHO (II/III/IV) | Mean | 83.2/90/  68.8 | 75.9/85.8/  70.1 | 77.1/78.3/  69.6 |
|  | MR | 49.7/55.1/  34.2^**^ | 45.5/59/  36.3^**^ | 48/50.7/  38.6 |
|  | p | .005 | .007 | .153 |
| New/ Re | Mean | 81.1/71.3 | 76.9/69.2 | 77.2/61.5 |
|  | MR | 49/31.5^*^ | 48/37.5 | 48.6/33.6 |
|  | p | .026 | .185 | .052 |

|  |  | SF | RF | QL |
| --- | --- | --- | --- | --- |
| Age  (<50y/≥50y) | Mean | 72.8/63.3 | 81.6/66.7 | 59.4/45.8 |
|  | MR | 50.2/40.4 | 50.6/39.9^*^ | 50/38.1^*^ |
|  | p | .079 | .045 | .035 |
| Sex (F/M) | Mean | 69.1/69.3 | 78.1/74.6 | 52/55.7 |
|  | MR | 45.1/47.4 | 47.5/45.9 | 43/47 |
|  | p | .686 | .751 | .484 |
| Location (Sup/  Sub) | Mean | 70/64.1 | 76.4/73.1 | 55.3/48.1 |
|  | MR | 47.5/40.2 | 46.7/45.4 | 46.5/39.4 |
|  | p | .346 | .866 | .361 |
| Location (L/R) | Mean | 70.3/68.9 | 77.6/75.4 | 56/54 |
|  | MR | 39.7/37.6 | 40.9/36.7 | 37.6/37.4 |
|  | p | .670 | .379 | .974 |
| Cognition (N/Ab) | Mean | 70/68.3 | 78.2/70.8 | 57.7/44.6 |
|  | MR | 46.3/45 | 47.5/40.6 | 47.8/35.4 |
|  | p | .840 | .258 | .057 |
| KPS  (<80/≥80) | Mean | 55.8/73.7 | 48.3/84.9 | 29.4/61.7 |
|  | MR | 28.9/46.7^**^ | 22.8/48.7^**^ | 21.5/48.1^**^ |
|  | p | .003 | .000 | .000 |
| WHO (II/III/IV) | Mean | 76.2/77.5/58.3 | 80.5/82.5/  66.2 | 58.1/66.7/  40.6 |
|  | MR | 52.2/51.6/  33.7^**^ | 49.4/51.4/  36.7^*^ | 47.8/55.9/  32.5^**^ |
|  | p | .004 | .036 | .002 |
| New/ Re | Mean | 71.9/52.6 | 77.6/65.4 | 56.7/39.7 |
|  | MR | 49.1/30.8^*^ | 48.2/36 | 47.7/32.3^*^ |
|  | p | .019 | .100 | .047 |

|  |  | FA | NV | PA |
| --- | --- | --- | --- | --- |
| Age  (<50y/≥50y) | Mean | 31.6/38.1 | 14.3/13.8 | 28.4/28.1 |
|  | MR | 43.1/52 | 46.7/46.2 | 46.3/46.8 |
|  | p | .119 | .914 | .930 |
| Sex (F/M) | Mean | 39.1/31 | 16.7/12.6 | 29.5/27.5 |
|  | MR | 52/43.1 | 49.5/44.7 | 47.9/45.6 |
|  | p | .114 | .321 | .681 |
| Location (Sup/Sub) | Mean | 34/34.2 | 11.6/29.5 | 27.4/33.3 |
|  | MR | 46.4/46.9 | 44.2/60.4 | 46/49.5 |
|  | p | .959 | .018 | .656 |
| Location (L/R) | Mean | 33.3/34.9 | 8.9/12.9 | 30.2/25.8 |
|  | MR | 37/39.6 | 36.5/39.9 | 41.7/36.2 |
|  | p | .602 | .410 | .260 |
| Cognition (N/Ab) | Mean | 33.7/33.9 | 13.6/15 | 27/30.8 |
|  | MR | 45.8/46.7 | 45.9/46.4 | 45.6/47.5 |
|  | p | .888 | .933 | .766 |
| KPS  (<80/≥80) | Mean | 60/25.7 | 30.8/9.4 | 45.8/22.9 |
|  | MR | 65.2/35.4^**^ | 49.1/40.5 | 53/39.2^*^ |
|  | p | .000 | .111 | .023 |
| WHO (II/III/IV) | Mean | 31.4/20.6/46.4 | 14.8/6.7/19.1 | 28.1/16.7/36.8 |
|  | MR | 41.7/29.1/57.7 | 44.7/35.9/50.6 | 43.5/33.3/53.4 |
|  | p | .000 | .063 | .015 |
| New/ Re | Mean | 32.6/42.7 | 14.4/12.8 | 28.3/28.2 |
|  | MR | 44.5/58.6 | 46.4/47.2 | 45.8/50.7 |
|  | p | .075 | .911 | .528 |

|  |  | SL | AP |
| --- | --- | --- | --- |
| Age  (<50y/≥50y) | Mean | 16.4/30.5 | 19.9/28.6 |
|  | MR | 42.7/52.7^*^ | 43.6/51.3 |
|  | p | .047 | .140 |
| Sex (F/M) | Mean | 24.8/19.9 | 29.5/19.3 |
|  | MR | 47.8/45.7 | 51.3/43.5 |
|  | p | .665 | .136 |
| Location (Sup/Sub) | Mean | 21.9/20.5 | 22.4/28.2 |
|  | MR | 46.9/44 | 45.8/51.1 |
|  | p | .681 | .464 |
| Location (L/R) | Mean | 16.7/25.8 | 16.7/26.5 |
|  | MR | 35.9/40.4 | 35.3/40.8 |
|  | p | .318 | .232 |
| Cognition (N/Ab) | Mean | 20.2/26.7 | 24.4/20 |
|  | MR | 44.7/50.6 | 47.1/42 |
|  | p | .313 | .400 |
| KPS  (<80/≥80) | Mean | 38.3/15.6 | 41.7/17.7 |
|  | MR | 53/39.2^*^ | 55.9/38.3^**^ |
|  | p | .011 | .002 |
| WHO (II/III/IV) | Mean | 20/13.3/29.4 | 22.9/16.7/29.4 |
|  | MR | 43.7/37.2/50.9 | 43.8/39.3/49.7 |
|  | p | .092 | .271 |
| New/ Re | Mean | 21.9/20.5 | 23.2/23.1 |
|  | MR | 47/43.6 | 46/49.4 |
|  | p | .625 | .644 |
